# Increased CSF Levels of Phosphorylated Neurofilament Heavy Protein following Bout in Amateur Boxers

**DOI:** 10.1371/journal.pone.0081249

**Published:** 2013-11-15

**Authors:** Sanna Neselius, Henrik Zetterberg, Kaj Blennow, Jan Marcusson, Helena Brisby

**Affiliations:** 1 Department of Orthopaedics, Sahlgrenska University Hospital, Gothenburg, Sweden; 2 Institution for Clinical Sciences, The Sahlgrenska Academy at University of Gothenburg, Gothenburg, Sweden; 3 Clinical Neurochemistry Laboratory, Department of Psychiatry and Neurochemistry, Sahlgrenska University Hospital, Gothenburg, Sweden; 4 Inst. of Neuroscience and Physiology, The Sahlgrenska Academy at University of Gothenburg, Gothenburg, Sweden; 5 Geriatric Section, University Hospital in Linköping, Linköping, Sweden; 6 Institution of Clinical and Experimental Medicine, Linköping University, Linköping, Sweden; University of Cambridge, United States of America

## Abstract

**Introduction:**

Diagnosis of mild TBI is hampered by the lack of imaging or biochemical measurements for identifying or quantifying mild TBI in a clinical setting. We have previously shown increased biomarker levels of protein reflecting axonal (neurofilament light protein and tau) and glial (GFAP and S-100B) damage in cerebrospinal fluid (CSF) after a boxing bout. The aims of this study were to find other biomarkers of mild TBI, which may help clinicians diagnose and monitor mild TBI, and to calculate the role of *APOE* ε4 allele genotype which has been associated with poor outcome after TBI.

**Materials and Methods:**

Thirty amateur boxers with a minimum of 45 bouts and 25 non-boxing matched controls were included in a prospective cohort study. CSF and blood were collected at one occasion between 1 and 6 days after a bout, and after a rest period for at least 14 days (follow up). The controls were tested once. CSF levels of neurofilament heavy (pNFH), amyloid precursor proteins (sAPPα and sAPPβ), ApoE and ApoA1 were analyzed. In blood, plasma levels of Aβ42 and ApoE genotype were analyzed.

**Results:**

CSF levels of pNFH were significantly increased between 1 and 6 days after boxing as compared with controls (p<0.001). The concentrations decreased at follow up but were still significantly increased compared to controls (p = 0.018). CSF pNFH concentrations correlated with NFL (r =  0.57 after bout and 0.64 at follow up, p<0.001). No significant change was found in the other biomarkers, as compared to controls. Boxers carrying the *APOE* ε4 allele had similar biomarker concentrations as non-carriers.

**Conclusions:**

Subconcussive repetitive trauma in amateur boxing causes a mild TBI that may be diagnosed by CSF analysis of pNFH, even without unconsciousness or concussion symptoms. Possession of the *APOE* ε4 allele was not found to influence biomarker levels after acute TBI.

## Introduction

Sports-related mild traumatic brain injury (TBI) is a common health issue at emergency departments [1] and there is growing awareness by medical professionals, trainers and athletes about the acute and long-term consequences after repeated mild TBIs. Diagnosis of mild TBI is hampered by the lack of imaging or biochemical measurements for identifying or quantifying mild TBI in a clinical setting.

We have recently shown in the same cohort, that repeated subconcussive trauma in amateur boxing (with head guard) causes a mild TBI, diagnosable by elevation of the brain injury biomarkers Neurofilament Light Protein (NFL), Glial Fibrillary Acidic Protein (GFAP), Total-tau (T-tau) and S-100B in the cerebrospinal fluid (CSF) [2]. In CSF, these biomarkers were elevated in >80% of the boxers between 1 and 6 days after bout, even without loss of consciousness or symptoms of a concussion. In most of the boxers the biomarker concentrations were normalized after 2 weeks of rest, but 20% still had significantly higher concentrations of NFL and GFAP than the matched control group. This study also showed that NFL concentrations correlated to the amount of head trauma but no correlation was found for GFAP, T-tau or S-100B [2]. In the same cohort, also brain injury biomarkers in blood were analyzed, where plasma T-tau was elevated directly after an amateur boxing bout (with head guard) although the results did not correlate with the CSF biomarkers [2], [3]. These findings indicate that biomarkers in CSF and blood may be used to diagnose, grade and monitor mild TBI.

Other biomarkers may also be useful in detecting mild TBI.

### Phosphorylated Neurofilament-Heavy Chain (pNFH)

Neurofilaments (NF) are exclusively found in neurons. As they are mainly involved in maintaining neuronal shape and size and conduction of nerve impulses along the axons [4], increased concentrations of NF proteins in CSF reflect axonal damage. NFH (Neurofilament-heavy chain) is one of the main three subunits of neurofilaments. The others are neurofilament-light chain (NFL) and neurofilament-medium chain (NFP). NFL, but not NFH, has previously been investigated in amateur boxers where increased concentrations were seen [2], [5]. NFH is mainly a phosphorylated protein, pNFH [4]. CSF levels of pNFH are elevated in axonal disorders such as amyotrophic lateral sclerosis (ALS) [6] and multiple sclerosis (MS) [7]. Increased serum concentrations of pNFH are also elevated after severe TBI, reflecting blood-brain-barrier dysfunction [8]. To the best of our knowledge, pNFH in CSF has not been analyzed in human after TBI, but an experimental study on rats showed elevated concentrations in serum both after spinal cord injury and moderate to severe TBI [9]. In this study, rats with spinal cord injury expressed a biphasic response, with an initial sharp peak after 16 h and a second less pronounced increase after 3 days. After TBI, increased levels of pNFH were also detected, but not as strong and with shorter time course (at 12 h and 2 days) compared to spinal cord injured rats. The first peak was believed to derive from acutely damaged axons and the second from secondary axonal degeneration.

### Amyloid Precursor Proteins (APP)

APP are expressed in neurons and have neurotrophic functions in promoting neuronal survival after axonal damage [10]. When APP is cleaved by β-secretase, β-sAPP and the N-terminus of Aβ is created with the formation of the Aβ – isoforms Aβ N-38, Aβ N-40, Aβ N-42, Aβ 1-40 and Aβ1-42. When APP is cleaved by α-secretase, α-sAPP is generated and Aβ formation prevented [11]. Deposits of various amyloid-β proteins are a hallmark of Alzheimer's Disease and TBI is considered a risk factor for the disease [12]. Low CSF concentrations of the aggregation-prone 42 amino acid isoform of Aβ (Aβ42), due to deposition of protein in plaques, have been observed during development of AD [13] and following TBI [14]. One human study showed that CSF levels of Aβ -40 and Aβ1-42 decline the first 5 days after severe TBI (GCS<8) [15]. Another study analyzing ventricular CSF following severe TBI showed increased Aβ1-42, α-sAPP and β-sAPP during the first week, although the plasma concentrations of Aβ1-42 remained unchanged [16]. The authors' explanation for this was that TBI induces APP-processing and Aβ formation, which eventually leads to Aβ aggregation.

### Apolipoproteins

Apolipoproteins are lipid transporters with six different subclasses (A, B, C, D, E and H). Apolipoprotein A1 (ApoA1) is the major protein component of high-density lipoprotein in plasma. In the CNS, ApoA1 may be a marker of neural degeneration and increased CSF concentrations have been seen in patients with AD, Parkinson's disease and multiple sclerosis [17]. In the CNS, apolipoprotein E (ApoE) is expressed and secreted by glia cells and neurons where it acts as a ligand for neuronal receptors and distributes cholesterol and phospholipids to injured neurons after injury [18]. ApoE plays a key role in the development of AD, where it is believed to promote plaque development. Reduced levels of ApoE are seen in AD [19]. A human study on severe TBI with Glascow Coma Scale (GCS) <8 found decreased concentrations of ApoE in CSF compared to controls the first 5 days post trauma. The concentrations of ApoA1 remained unchanged. The authors believed that ApoE is consumed by neurons as a response to acute injury [15]. To our knowledge, CSF ApoA1 and ApoE have not been studied in relation to MTBI.

### Apolipoprotein E genotype (APOE)

The *APOE* gene is located on chromosome 19 and has three common alleles (ε2, ε3 and ε4) where the presence of *APOE* ε4 is associated with unfavorable outcome after TBI [20] and chronic traumatic encephalopathy in boxers [21]. Presence of the *APOE* ε4 is a well-known risk factor for AD [22]. TBI is also a risk factor for AD [23], [24], [25]. The presence of *APOE* ε4 in combination with TBI is suggested to additionally increase the risk for developing AD [23], [26].

Although the available evidence suggests that pNFH, APPs and apoliproteins are affected by TBI, most of these markers have only been investigated after severe TBI or in animal models. To the best of our knowledge, none of these markers have been investigated in CSF after a mild TBI in the absence of obvious symptoms. The primary aim of the present study was to investigate whether pNFH, amyloid precursor proteins, or apolipoproteins in CSF and/or blood are useful biomarkers in the diagnosis and monitoring of mild TBI, The study was performed in the same cohort of boxers as our previous biomarker studies [2], [3] which enabled us to correlate the findings with previously detected biomarkers. The secondary aim was to analyze whether the *APOE* ε4 genotype influences biomarker concentrations after brain trauma.

## Materials and Methods

### Study design and population

The study was designed as a prospective follow-up study. Thirty amateur boxers competing at elite level were compared to 25 healthy, age-matched controls. All boxers had completed at least 45 bouts. This number was based on the regulation of the Swedish National Boxing Federation demanding an examination with MRI, CT or EEG every 50 bouts. The control group consisted of friends or relatives to the boxers, aiming to get control subjects with a similar social background and education level to the boxers. Exclusion criteria were athletes at senior elite level in sports where head trauma may occur, e.g., soccer, ice hockey and martial arts.

### Ethical Issues

The Regional Ethical Review Board in Linköping, Sweden approved the study. Written informed consent was obtained from all participants.

### Questionnaire design and neurological examination

All participants filled in a questionnaire about medical history, medication, education, present occupation, information about previous concussions and quantification of alcohol and drug intake. Previous sports career was reported, to identify those who had trained in sports with risk of TBI. The questionnaire included a 10-question survey regarding previous and current symptoms of head and neck injuries based on a previous study [27]. The number of head and neck symptoms that had worsened over the last 5–10 years was added in a score. The boxers reported about their boxing career; fighting record, number of knock-out (KO) losses, number of Referee Stopping Contest losses due to several hard punches to Head (RSC-H), present weight class, duration of career, age at career start and age at first bout. Boxers reported the total number of bouts the last week prior testing (1–3 bouts) and estimated these bouts as easy (1), intermediate (2) or tough (3). Three experts independently graded the boxers regarding their “boxing type” as 1 to 5 (low to high risk), considering head trauma during total boxing career. These factors were added and named “Boxing Exposure”. The aim was to calculate the total TBI risk prior to testing.

All participants underwent a neurological examination. The neurological examination protocol included anamnestic questions about concussion symptoms, a general somatic status (general condition, examination of mouth and throat, heart, blood pressure, abdominal palpation, peripheral circulation and skin status) and neurological status (orientation, alertness, speech function, cranial nerves 1–12, motor skills, balance, coordination, gate, sensibility testing and testing of reflexes). Magnetic resonance imaging (MRI) of the brain and neuropsychological testing (including among others short and long time memory, mental speed, recollection and cognitive testing) were performed in all participants without the findings of any structural injuries (hemorrhages, subdural hematomas) or other major pathological findings observed. Detailed results of these investigations will be presented in a separate paper.

### CSF sample collection

The lumbar puncture was performed at daytime, between 10 a.m. and 3 p.m., with the study objects in sitting position or lying on one side. For the first 18 participants a Quincke Type Point spinal needle (22 Gauge) were used, but since a few of the study participants suffered from postspinal headache, the needle was changed to a Sprotte (24 Gauge). Thereafter no more postspinal headache occurred. For each study participant 5 to 10 ml CSF was collected in a polypropylene tube (Sarstedt, Nümbrecht, Germany), gently mixed to avoid gradient effects, aliquoted and stored at −80°C pending analysis. LPs were performed twice in the boxers: First LP 1–6 days after a bout (test A) and the second without exposure to bouts or training with blows to head for at least 14 days (test B). The control subjects underwent one LP.

### Blood sample collection

Blood was collected by venipuncture into whole blood and gel-separator tubes. The samples were centrifuged within 20–60 minutes, aliquoted and stored at −80°C pending analysis. Venipuncture were performed twice in the boxers: First 1–6 days after a bout (test A) and the second without exposure to bouts or training with blows to the head for at least 14 days (test B). The control subjects underwent one venipuncture.

### CSF biomarker analyses

CSF NFH was analyzed using a sandwich ELISA (Abnova, Walnut, CA, USA) by a person blinded to the experimental groups. CSF AβX-38, AβX-40 and AβX-42 levels were measured by the electrochemiluminescence technology using the MS6000 Human Abeta 3-Plex Ultra-Sensitive Kit, while β-secretase cleaved soluble APP (sAPP-β) and α-secretase cleaved soluble APP (sAPP-α) were measured using the MS6000 Human sAPPalpha/sAPPbeta Kit (Meso Scale Discovery, Gaithersburg, Maryland, USA), as decribed previously [28]. CSF levels of ApoE and ApoA1 were measured using the MILLIPLEX MAP Human Apolipoprotein Panel (Millipore Corporation, Billerica, MA, USA) in a Bio-Plex instrument (Bio-Rad Laboratories, Inc., Herts, UK). Quantification of Aβ1-42 in plasma was performed by single molecule digital ELISA, as described previously in detail [29].

### APOE genotyping

*APOE* (gene map locus 19 q13.2) genotyping was performed using TaqMan® Allelic Discrimination technology (Applied Biosystems, Foster City, CA). Genotypes were obtained for the two SNPs that are used to unambiguously define the ε2, ε3, and ε4 alleles (rs7412 and rs429358).

### Statistics

Statistical analysis was carried out with SPSS 16.0. Comparisons between groups were performed using the non-parametric Mann-Whitney U-test, as some of the biomarker concentrations were skewed. Comparison between test A and test B were performed with Related Samples Wilcoxon Signed Rank Test. Correlation analyses were performed with Spearman two-tailed test.

## Results

All participants were included in the CSF biomarker analysis 1 to 6 days after a boxing bout (N = 30). Two participants with complications after the lumbar puncture (back pain and headache, respectively) and two without complications declined follow-up (N = 26 at follow-up).

### Baseline factors and neurological examination

Baseline factors about medical and social history and the 10-question survey were similar between boxers and controls and detailed results of the questionnaire have been published previously [2]. The mean age was 22 in both groups and they had the same education level. The majority were male with 2 females in the boxer group and 5 in the control group. The earlier concussion rate in the groups was similar but 24% of the controls had played other sports, such as ice hockey and soccer (boxing excluded), where head injury can occur, for more than 10 years. This number was 0% for the boxers. Only one of the boxers reported concussion-related symptoms after bout (in this case headache) at the clinical examination, but the medical and neurological examination was normal in all subjects, with GCS 15.

### CSF levels of pNFH

The results of pNFH are presented in Table 1. There was a significant difference between boxers and controls both at test A (1 to 6 days; p<0.001) and B (≥14 days; p = 0.02). The highest concentration was possessed by one of the controls, 1265 ng/L, who differed substantially compared to the other controls (range 27–206 ng/L). This control was a 19-year-old man, who had played soccer and ice hockey for 16 years. In fact, he had also boxed for one year at age 12. At the time of the test he was an active ice hockey player. He had suffered from a sports-related concussion once, during an ice hockey game five years earlier. The alcohol intake was once a week, which was more than average. He also showed the highest value for AβN-42 with 1119 ng/L. In an earlier presented paper on this study cohort, this control also had the highest concentration of P-Tau but normal NFL concentrations [2]. We offered this control a follow up, but he declined. At test B, the only boxer reporting sequelae (headache) after the bout possessed the highest NFL concentration (1503 ng/L).

**Table 1:** Biomarker concentrations in cerebrospinal fluid and plasma.^4^

Marker	Boxer Test A^1^	Boxer Test B^2^	Controls	P-value		
	N = 30	N = 26	N = 25^4^	A vs C	A vs B	B vs C
	Md(range)SD ng/L	Md(range)SD ng/L	Md(range)SD ng/L			
**pNFH** 3	163(49–562)117	68(23–1503)298	33 (27–1265) 251	0.000	0.018	0.018
**AβN38**	1541(715–2890)606	1538(566–2733)578	1578 (925–3066) 582	0.618	0.622	0.851
**AβN40**	7211(4017–11100)1919	7290(3315–10979)1836	7588(4887–10610)1658	0.993	0.501	0.638
**AβN42**	589(276–1380)268	606(227–1002)216	641(378–1119)214	0.846	0.657	0.468
**Aβ _1-42_** 4	12(4–27)5	12(0.0–20)4	12(0.7–19) 4	0.906	0.288	0.861
**sAPPα**	635(319–1122)189	666(227–1048)209	587 (359–988) 180	0.654	0.218	0.442
**sAPPβ**	207(110–405)80	220(54–508)97	197(116–406) 78	0.565	0.334	0.462
**ApoE**	4597(2659–9577)1647	4411(2751–7859)1321	3977(2131–7192)1432	0.128	0.534	0.109
**ApoA1**	1936(834–4673)1018	2435(1273–5589)986	2155(1033–5853)1247	0.710	0.006	0.221

1 Test A: 1–6 days after last bout;

2 Test B: No boxing for at least 14 days.

3 According to pNFH, the result from one of the controls was destroyed.

4 Aβ **_1-42_** was collected from plasma.

There was no correlation between pNFH and boxing exposure (r = 0.251).

### CSF APP, ApoA1, ApoE and Aβ-isoforms

There was a slight increase in median ApoA1between timepoint A (1 to 6 days) and B (≥14 days) for the boxers (p = 0.006), but there was no significant difference between boxers and controls. For the biomarkers sAPPα, sAPPβ and the Aβ-isoforms Aβ N-38, Aβ N-40 and Aβ N-42, no differences were seen between the different timepoints for the boxers, or between the boxing and control groups. The results are presented in detail in Table 1.

### Serum Aβ

No significant differences were seen between the groups at any of the two time-points (table 1).

### Correlation of pNFH with previously analyzed biomarkers

The results from the analyses in the present study were compared to previously analyzed biomarkers on the same study cohort [2], [3]. This showed that pNFH levels correlated with CSF NFL (r = 0.589, p = 0.002) [2]. No correlations were found between pNFH and the other previously analyzed biomarkers CSF GFAP, Total-tau, S-100B [2] or plasma Tau [3].

### APOE genotyping

Twelve of the 30 boxers were *APOE* ε4 carriers vs 7 of the controls. When comparing the biomarker concentrations presented in this paper and the CSF biomarkers NFL, GFAP, P-tau and S-100β [2] for the boxers that were carriers vs non carriers, there were no significant differences between the groups. The p-values ranged from 0.094 (serum Aβ) to 0.966.

## Discussion

The purpose of the study was to investigate whether pNFH, amyloid precursor proteins, or apolipoproteins in CSF and/or blood are useful biomarkers in the diagnosis and monitoring of mild TBI to analyze whether the *APOE* ε4 genotype influences biomarker concentrations after brain trauma. The study was performed in the same cohort of boxers as our previous biomarker studies which enabled us to correlate the findings with previously detected biomarkers.

pNFH in CSF increases after a amateur boxing bout (with head guard) and does not normalize within 14 days of rest, even though none of the boxers had suffered from KO and only one of the boxers had concussion symptoms (headache) after bout. None of the CSF biomarkers APP, Aβ isoforms, ApoA1, Apo E and serum Aβ demonstrated any pathological concentrations.

The CSF pNFH levels significantly correlated with earlier analyzed CSF NFL [2], but in contrast to NFL, pNFH did not correlate with boxing exposure (the amount of hits to the head). The presence of NFL and pNFH in the CSF reflect axonal damage but their release curves after acute brain trauma are still unknown.

This study suggests that the CSF biomarkers APP, Aβ isoforms, Apo E and serum Aβ are not useful in the diagnosis or monitoring of mild TBI or early stages of chronic TBI. However, ApoA1 significantly increased between test A and B for the boxers, although there was no significant difference between boxers and controls. This is interesting, since ApoA1 is believed to be a marker of neural degeneration and increased CSF concentrations have been seen in patients with neurodegenerative disorders such as AD, Parkinson's disease and multiple sclerosis [17].

The lack of association between mild TBI and the amyloid-related biomarkers is also interesting given that earlier studies have established a relationship between severe TBI and abnormal amyloid metabolism [14], [15], [16]. One reason for the difference could be that the head blows received by most boxers in our study did not impact their brains as much as in the other studies. Here, none of the boxers had been knocked-out and only one complained of concussion symptoms (headache). Another potential reason is the variability in the head trauma exposure among the boxers, which could make it hard to establish weak associations. In any case, our data suggest that markers of axonal injury are more sensitive than amyloid-related markers to detect acute brain changes in mild TBI.

A recent study on severe TBI showed higher concentrations of S-100β in the CSF of *APOE* ε4 carriers versus non carriers [30]. Our results could not support these findings. *APOE* ε4 carrier status did not influence the CSF concentrations of pNFH. Furthermore, we did not find any relationship between *APOE* ε4 carrier status and any of the earlier published concentrations of NFL, GFAP, T-tau or S-100β in this study group [2], although there was a trend towards lower serum Aβ concentrations in the *APOE* ε4-positive group.

The biomarkers identified by this study and our earlier published studies with the same cohort [2], [3] show that a mild TBI can be detected even if the clinical and/or anamnestic criteria for a mild TBI are not fulfilled. This is important, as these biomarkers may assist clinicians in determining when to recommend an athlete to return to play after a head injury.

Ice hockey and soccer are examples of popular sports with high risk for concussions and it is known that coaches and players do not always follow the return-to-play guidelines [31], [32], [33], which allows return to play within a week in the absence of concussion symptoms. Ice hockey has a clinical diagnostic MTBI incidence of 5.0–5.4% [34], [35] and soccer 6.4% per match [34]. According to our study and previously published studies [2], [5], the brain needs longer time for full recovery after head trauma than previously known.

In conclusion, pNFH significantly increased between 1 and 6 days after an amateur boxing bout (with head guard). After a rest period of at least 14 days, the concentrations decreased but were still significantly elevated compared to controls. *APOE* ε4-carrier status did not influence biomarker concentrations after acute TBI. Thus, sub-concussive repetitive trauma in amateur boxing causes a mild TBI, and without proper rest there might be an increased risk for long-term consequences.

The results of this study, and our previous studies in the same cohort, provide insight into the pathophysiology of sports-related mild TBI. Moreover, they suggest that pNFH, NFL, GFAP, T-Tau and S-100β [2] may be valuable CSF biomarkers in the detection and evaluation of mild TBI.
